# Predicting Falls in Parkinson Disease: What Is the Value of Instrumented Testing in OFF Medication State?

**DOI:** 10.1371/journal.pone.0139849

**Published:** 2015-10-07

**Authors:** Martina Hoskovcová, Petr Dušek, Tomáš Sieger, Hana Brožová, Kateřina Zárubová, Ondřej Bezdíček, Otakar Šprdlík, Robert Jech, Jan Štochl, Jan Roth, Evžen Růžička

**Affiliations:** 1 Department of Neurology and Centre of Clinical Neuroscience, First Faculty of Medicine and General University Hospital in Prague, Charles University in Prague, Prague, Czech Republic; 2 Department of Cybernetics, Faculty of Electrical Engineering, Czech Technical University in Prague, Prague, Czech Republic; 3 Department of Control Engineering, Faculty of Electrical Engineering, Czech Technical University in Prague, Prague, Czech Republic; 4 Department of Kinanthropology, Charles University in Prague, Prague, Czech Republic; 5 Institute of Neuroradiology, University Medicine Goettingen, Goettingen, Germany; 6 Department of Health Sciences, University of York, York, United Kingdom; Karolinska Institute, SWEDEN

## Abstract

**Background:**

Falls are a common complication of advancing Parkinson's disease (PD). Although numerous risk factors are known, reliable predictors of future falls are still lacking. The objective of this prospective study was to investigate clinical and instrumented tests of balance and gait in both OFF and ON medication states and to verify their utility in the prediction of future falls in PD patients.

**Methods:**

Forty-five patients with idiopathic PD were examined in defined OFF and ON medication states within one examination day including PD-specific clinical tests, instrumented Timed Up and Go test (iTUG) and computerized dynamic posturography. The same gait and balance tests were performed in 22 control subjects of comparable age and sex. Participants were then followed-up for 6 months using monthly fall diaries and phone calls.

**Results:**

During the follow-up period, 27/45 PD patients and 4/22 control subjects fell one or more times. Previous falls, fear of falling, more severe motor impairment in the OFF state, higher PD stage, more pronounced depressive symptoms, higher daily levodopa dose and stride time variability in the OFF state were significant risk factors for future falls in PD patients. Increased stride time variability in the OFF state in combination with faster walking cadence appears to be the most significant predictor of future falls, superior to clinical predictors.

**Conclusion:**

Incorporating instrumented gait measures into the baseline assessment battery as well as accounting for both OFF and ON medication states might improve future fall prediction in PD patients. However, instrumented testing in the OFF state is not routinely performed in clinical practice and has not been used in the development of fall prevention programs in PD. New assessment methods for daylong monitoring of gait, balance and falls are thus required to more effectively address the risk of falling in PD patients.

## Introduction

Falls are a common and disabling complication of advancing Parkinson’s disease (PD). In various studies, 35 to 90% of patients have reported at least one fall per year and two thirds of patients are recurrent fallers [1]. Several retrospective [2–4] as well as prospective [5–7] studies have sought to determine risk factors or predictors of falls in PD with inconsistent results. The best clinical predictors of future falls in PD reported in prospectively designed studies appear to be a history of falls [6–10], increased disease severity [5, 9, 11], the presence of freezing of gait (FOG) [8, 12], fear of falling [13, 14], poor balance [8, 12, 15, 16] and reduced mobility [12, 17, 18]. A few studies have also suggested that instrumented measures such as leaning balance in the coordinated stability test, cadence, walking speed and mediolateral head motion could discriminate between prospectively identified PD fallers and non-fallers [8, 19].

In daily life, patients with advanced PD commonly sustain levodopa-induced motor fluctuations and dyskinesias. It is unclear however whether the current motor state affects the likelihood of falling. It has been suggested that falls occur rather in the ON medication state when patients are more mobile [20], while others have argued that falls also occur in the OFF state due to poor motor performance [21]. Examination in both the ON and OFF states may help to elaborate fall risk in relation to medication state [22]. However, only one prospective study to date has examined the responsiveness and predictive validity of motor parameters tested in both OFF and ON medication states, suggesting that OFF medication performance provides more accurate prediction of future falls [17].

In the present study, we aimed to determine if the medication state affects the value of gait and balance tests in predicting future falls. In addition, we aimed to identify levodopa-responsive factors contributing to falls in prospectively identified PD fallers.

## Patients and Methods

### Ethics statement

The research protocol was approved by the local research Ethics Committee of the General University Hospital, Prague (the approval number: 120/08) in accordance with the Declaration of Helsinki. All participants provided signed, informed consent before entering the study.

### Subjects

Forty-five patients with PD diagnosed according to the United Kingdom PD brain bank criteria were included in the study [34 men, 11 women; mean age 67.2 (SD 7.4, range 49–81) years; PD duration 10.2 (SD 3.4, range 6–20) years; mean Hoehn and Yahr (HY) stage 2.6 (range 2–3)]. Patients were eligible if they were able to ambulate independently without a walking aid in both ON and OFF medication states, did not report daily falls and were without a history or clinical signs of any other neurological, orthopedic or sensory disorders that could interfere with balance and gait. Disease duration of 6 or more years and a clear beneficial response to levodopa dose lasting at least two hours was required, excluding patients with random motor fluctuations. None of the PD patients had undergone brain surgery. A stable anti-parkinsonian medication was required for at least 4 weeks before the baseline assessment. All patients were treated with levodopa, in 32/45 combined with a dopamine agonist and in 16/45 with entacapone (see main clinical characteristics of patients and controls in **Table 1**). Twenty-two healthy control subjects [13 men, 9 women; mean age 65.5 (SD 8.4, range 48–80) years] were recruited from the patients’ relatives and from hospital personnel not involved in the study. Subjects were considered eligible if they were able to ambulate independently without walking aids, did not report daily falls and were without a history or clinical signs of any disorders that could interfere with balance and gait.

**Table 1:** Main characteristics of PD patients and control subjects.

Parameters	PD (N = 45; M34, F11)	NC (N = 22; M13, F9)	p-value^a^
	Mean (SD)	Mean (SD)	
Age (yrs)	67.2 (7.4)	65.5 (8.4)	0.389
PD duration (yrs)	10.2 (3.4)	NA	NA
Hoehn and Yahr stage	2.6 (0.4)	NA	NA
Levodopa equivalent (mg)	1124.9 (400.1)	NA	NA
UPDRS-III OFF	28.4 (9.6)	NA	NA
UPDRS-III ON	16.8 (8.6)	NA	NA
GBS subscore OFF	5.2 (2.7)	NA	NA
GBS subscore ON	2.8 (2.1)	NA	NA
NMS-30	8.9 (4.1)	NA	NA
MoCA	24.2 (3.3)	26.6 (1.3)	0.002*
FAB	15.0 (2.4)	17.0 (0.9)	<0.001*
BDI-II	10.2 (5.9)	6.6 (7.1)	0.003*
STAI X1	36.9 (7.9)	31.1 (6.3)	0.001*
STAI X2	41.0 (8.3)	36.8 (8.4)	0.019
FES-I	11.9 (3.7)	8.0 (1.0)	<0.001*
ESSOT OFF	72.4 (10.2)	78.0 (7.1)	0.031
ESSOT ON	75.6 (7.6)		0.288
Gait speed OFF (m/s)	0.93 (0.14)	1.19 (0.11)	<0.001*
Gait speed ON (m/s)	1.02 (0.14)		<0.001*
Cadence OFF (steps/min)	113.2 (10.5)	109.1 (10.7)	0.208
Cadence ON (steps/min)	114.2 (10.7)		0.108
Stride time variability (CV%) OFF	3.4 (1.3)	2.0 (0.8)	<0.001*
Stride time variability (CV%) ON	2.3 (0.8)		0.126

a based on Wilcoxon exact test

*differences significant at the Holm- Bonferroni-corrected level of p < 0.05 (for 15 tests performed)

Same control values were used for comparisons with PD patient values obtained in OFF and ON conditions

*Abbreviations*: PD: Parkinson’s disease patients; NC: normal controls; SD: standard deviation; OFF: “off” medication state; ON: “on” medication state; UPDRS-III: Unified Parkinson’s Disease Rating Scale, Motor score; GBS: Gait and balance score; NMS-30: Non-motor symptom scale; MoCA: Montreal Cognitive Assessment; FAB: Frontal Assessment Battery; BDI-II: Beck Depression Inventory, Second Edition; STAI: State-Trait Anxiety Inventory (X1 = State anxiety, X2 = Trait anxiety); FES-I: Short Falls Efficacy Scale-International; ESSOT: Composite equilibrium score in Sensory organization test; CV: coefficient of variation; NA: not available

### Clinical assessment

Demographic information and a history of falls in the previous 6 months were obtained from all participants in a personal interview prior to gait and balance evaluation. All PD participants were evaluated in OFF and ON medication states within one examination day, further referred to as a baseline. They first underwent examination in a clinically defined OFF state in the morning following the withdrawal of all dopaminergic medications (48 h for dopamine agonists, 12 h for levodopa and COMT inhibitors) and then in the ON state following a dose of levodopa equivalent to 150% of their usual morning dose. In both OFF and ON states, patients were examined using the Unified Parkinson’s Disease Rating Scale (UPDRS) part II and III, from which the gait/balance subscore (GBS) was calculated as the sum of UPDRS-III items 27–31 and part IV, from which the presence of dyskinesia (UPDRS IV A ≠ 0) and dyskinesia subscore was calculated as the sum of UPDRS items 32 and 33 [7]. A test to provoke FOG was performed by rising from a chair, walking 5 m in a narrow space between two chairs, turning 180°, walking back and sitting down [23]. FOG and motor blocks were visually rated on a 0–1 scale. To ensure that the patient´s motor state remained stable in the ON state, UPDRS-III was checked again at the end of testing. In addition, neuropsychological testing was performed in the ON state, consisting of the Montreal Cognitive Assessment (MoCA) [24], Frontal Assessment Battery (FAB) [25], Beck Depression Inventory-Second Edition (BDI-II) and the State-Trait Anxiety Inventory (STAI X1 and X2) [26] according to test manuals or instructions. Patient participants were also asked to complete the Short Falls Efficacy Scale-International (FES-I) [27] and Non-motor Symptom Scale (NMS-30) [28] questionnaires. Control subjects were tested in a single session consisting of the FES-I questionnaire and neuropsychological assessment (MoCA, FAB, BDI-II, STAI).

### Instrumented evaluation of balance and gait

Balance and gait were assessed in all participants; in PD patients in both the OFF and ON medication states, within one examination day. Gait analysis was performed using portable inertial sensors (Xsens MTx; Enschede, the Netherlands) in the instrumented Timed Up and Go (iTUG) test extended from 3 m (traditional TUG test) to 7 m to provide enough steps for gait analysis according to Zampieri et al. [29]. Three walking trials were performed at self-paced walking speed and the following gait outcomes representing general mobility and gait variability were collected within the second trial: (1) Gait speed (m/s); (2) Cadence (steps/min) and (3) Stride time variability [the coefficient of variation (CV) of stride durations], which was chosen as a potential predictor of falls in PD patients [21]. Balance was evaluated by means of computerized dynamic posturography (The Smart Balance Master®; Neurocom, Clackamas, Oregon, USA) using a composite equilibrium score in the sensory organization test (ESSOT) [30]. Detailed instrumented evaluation of balance and gait is included as Supporting Information (**S1 File**).

### Follow up period

After the clinical and instrumented examination, the participants were followed for 6 months with fall incidence determined through fall diaries, with the instruction to record each fall and near fall as well as all circumstances and consequences of falls. Subjects were asked to return the diaries by post each month and they were contacted monthly by telephone to assure that all falls were documented. They were classified as fallers if they reported one or more falls by the end of the 6 month observation period and as non-fallers if they reported no falls in the same interval. The data from diaries were used to classify falls according to Maki et al. as well as Lach et al. [5]. Anti-parkinsonian as well as other medical treatment remained without any changes in all PD participants during the follow up period.

### Statistical analyses

The Exact Wilcoxon test [31] was used to compare parameters of patients and healthy subjects, and parameters of fallers and non-fallers. A paired t-test was used to compare parameters between ON and OFF medication states. The relationship between stride time variability and other parameters was assessed post-hoc using the Pearson correlation coefficient. Holm-Bonferroni correction was applied to address the problem of multiple testing.

To determine which patient parameters were associated with an increased probability of falling, a logistic regression model was built for each parameter independently and the predictive relevance of each parameter was assessed using the likelihood ratio test. A multiple logistic model was then sought to predict falling. Due to the rather limited number of patients in the present study, a limited number of predictors were included in the model. Therefore, a set of candidate predictors was chosen with consideration to those parameters previously identified to be associated with falling. Two sets of parameters were considered: clinical and instrumented, and two respective models were built based on these sets. Then, a final model was sought by combining the clinical and the instrumented models. To demonstrate model performance, Akaike information criterion (AIC) and Bayesian information criterion (BIC) were calculated and the receiver operating characteristic (ROC) curves were constructed. Data analyses were carried out in R [32].

## Results

All patient and control subjects completed the clinical, neuropsychological and posturographic assessments. Gait assessment was not completed in three PD patients in the OFF state due to their inability to walk the entire TUG distance independently. Therefore, we show gait data in the OFF state in 42 PD subjects. All PD patients were able to complete the gait task in the ON state. Baseline parameters of PD patients and controls are shown in **Table 1**. All subjects completed the 6-month follow-up period, in which 27 PD patients (60%) fell one or more times, while 18 patients did not fall. From the control group, four subjects (18%) fell and 18 did not (**S1 Table**).

**Table 2**shows comparison between the two prospectively identified subgroups of PD fallers (PD-F) and PD non-fallers (PD-NF), demonstrating differences between baseline parameters according to falling status. A higher retrospective fall record, HY stage, mean levodopa equivalent dose and greater fear of falling (FES-I) characterized PD-F compared to PD-NF. Also OFF (but not ON) state UPDRS-III and GBS scores were significantly higher in PD-F compared to PD-NF. PD-F also had more pronounced depressive symptoms according to BDI-II, corresponding to mild mood disturbance in 9/27 PD-F and 4/18 PD-NF, and borderline/moderate depression in 7/27 PD-F only. When analyzed separately, none of the individual BDI-II items significantly differed between fallers and non-fallers. Four patients reported FOG, and while all of them entered the PD-F group, the FOG parameter (item 14 of the UPDRS-II) did not discriminate between PD-F and PD-NF. Presence of dyskinesia was reported in 15/27 (56%) PD-F compared to 7/18 (39%) PD-NF, but this parameter did not discriminate between both groups. Global cognitive performance (MoCA), frontal function (FAB) and dyskinesia subscore were not related to falling status. Stride time variability in the OFF medication state was the only parameter of instrumented gait and balance analysis that significantly differed between PD-F and PD-NF (**Table 2**). Stride time variability in the OFF state larger than 3.25% (**Fig 1A**) predicted falls with a sensitivity of 96%, specificity of 83% and an AUC of 0.933.

**Fig 1:**
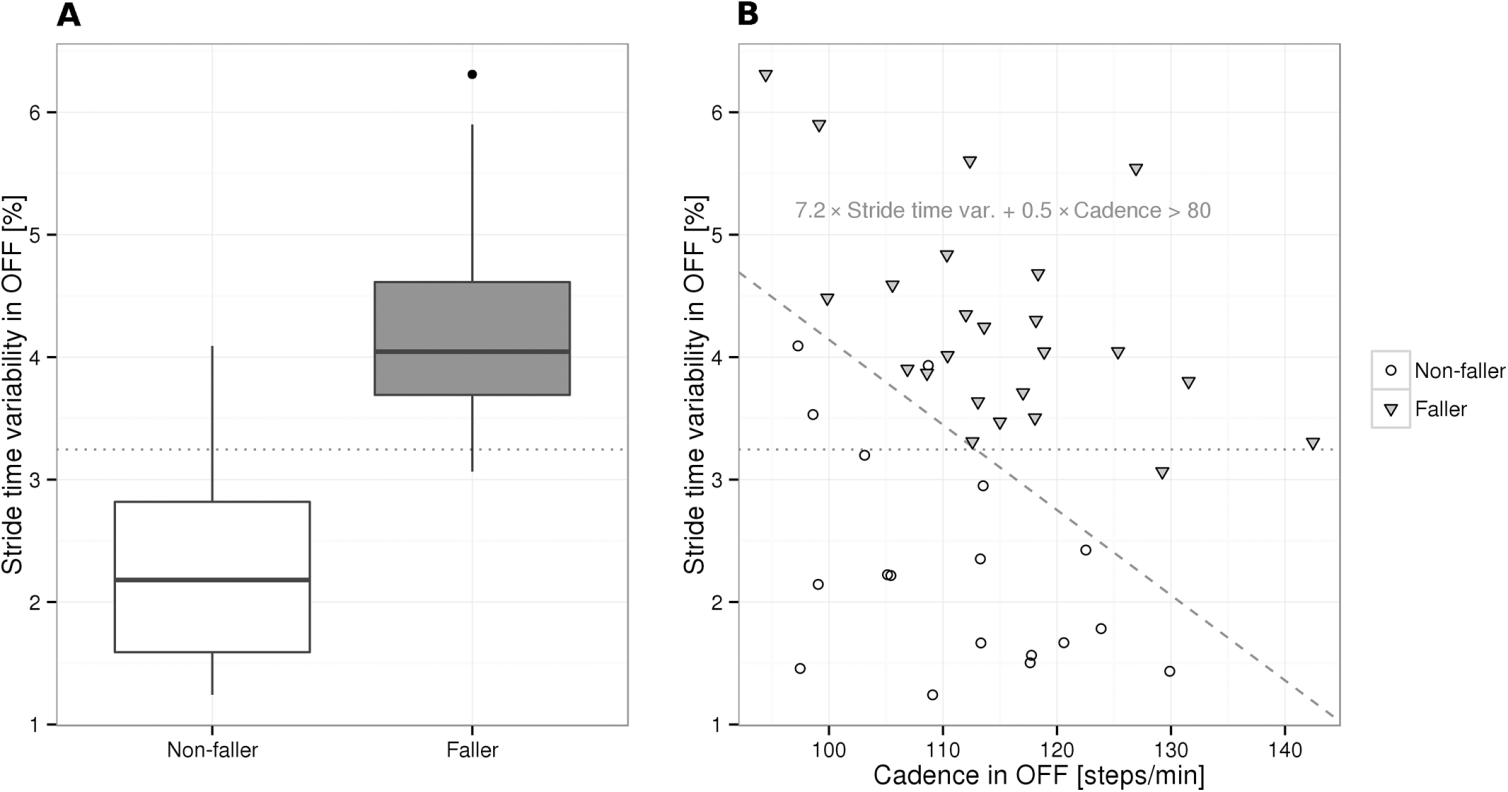
Comparison of PD fallers and non-fallers based on logistic regression models. A: PD fallers exhibited higher stride time variability compared with non-fallers. The dotted line shows the optimal logistic regression classification threshold of 3.25%. B: The multiple logistic regression model predicted fallers as patients having 7.2 × stride time variability in the OFF state + 0.5 × cadence in the OFF state greater than 80 (corresponding to the area above the dashed line). The dotted line shown in A is overlaid for visual comparison.

**Table 2:** Baseline parameters of PD fallers and PD non-fallers.

Domains and parameters	PD–F(N = 27; M20, F7)	PD–NF(N = 18; M14, F4)	Odds ratio^a^	95% CI	p-value^b^
	Mean (SD)	Mean (SD)			
**Demographic and disease specific parameters**					
Age (yrs)	65.6 (7.7)	69.7 (6.3)	0.92	(0.83, 100)	0.063
Falled in previous 6 m	n = 21 [77.8%]	n = 2 [11.1%]	28	(6, 213)	<0.001*
PD duration (yrs)	10.4 (4.0)	9.8 (2.3)	1.05	(0.88, 1.28)	0.571
Hoehn and Yahr stage	2.7 (0.3)	2.3 (0.4)	16.00	(3.01, 117.11)	<0.001*
Levodopa equivalent (mg)	1265 (430)	915 (232)	1.34^c^	(1.10, 1.74)^c^	0.002*
UPDRS-III OFF	31.7 (10.1)	23.5 (6.3)	1.31	(1.04, 1.26)	0.002*
UPDRS-III ON	18.6 (9.4) †	14.1 (6.5) †	1.08	(1.00, 1.18)	0.067
Presence of dyskinesia	n = 15 [55.6%]	n = 7 [38.9%]	1.96	(0.59, 6.86)	0.272
Dyskinesia subscore	0.63 (0.79)	0.56 (0.78)	1.13	(0.52, 2.61)	0.751
GBS OFF subscore	6.4 (2.5)	3.4 (1.9)	2.13	(1.42, 3.72)	<0.001*
GBS ON subscore	3.4 (2.3) †	1.8 (1.2) †	1.70	(1.15, 2.81)	0.005
FOG	0.6 (1.1)	0.3 (0.5)	1.57	(0.74, 4.45)	0.264
NMS-30	10.1 (3.9)	7.0 (3.8)	1.24	(1.05, 1.50)	0.009
FES-I	13.2 (3.9)	9.9 (2.3)	1.45	(1.14, 1.97)	<0.001*
**Cognition, anxiety parameters**					
MoCA	24.0 (3.7)	24.6 (2.4)	0.94	(0.78, 1.14)	0.544
FAB	14.7 (2.7)	15.6 (1.9)	0.85	(0.63, 1.10)	0.236
BDI-II	12.4 (6.2)	7.0 (3.7)	1.25	(1.08, 1.51)	<0.001*
STAI X1	38.6 (8.8)	34.3 (5.6)	1.08	(0.99, 1.19)	0.067
STAI X2	42.3 (9.5)	39.1 (5.6)	1.05	(0.98, 1.14)	0.195
**Instrumented balance and gait parameters**					
ESSOT OFF	70.9 (11.2)	74.7 (8.3)	0.96	(0.89, 1.02)	0.198
ESSOT ON	74.2 (8.4)	77.7 (5.9)	0.93	(0.84, 1.02)	0.121
Gait velocity OFF (m/s)	0.92 (0.14)	0.93 (0.14)	0.56	(0.01, 48.67)	0.797
Gait velocity ON (m/s)	1.02 (0.14) †	1.03 (0.15) †	0.77	(0.01, 54.96)	0.902
Cadence OFF (steps/min)	115.0 (10.8)	110.9 (9.9)	1.04	(0.98, 1.11)	0.201
Cadence ON (steps/min)	115.7 (11.8)	112.0 (8.6)	1.04	(0.98, 1.10)	0.240
Stride time variability (CV%) OFF	4.3 (0.9)	2.3 (0.9)	17.20	(4.31, 196.41)	<0.001*
Stride time variability (CV%) ON	2.6 (0.8) †	2.0 (0.8)	2.59	(1.13, 7.18)	0.024

^a^ odds ratio for falling related to the unit change in each parameter (in all PD patients)

^b^ based on likelihood ratio test in logistic regression model

^c^ unit change defined as 100mg of L-DOPA equivalent

*PD-F vs. PD-NF differences significant at the Holm-Bonferroni-corrected level of p < 0.05 (for 27 tests performed)

† ON vs. OFF differences significant at the Holm-Bonferroni-corrected level of p < 0.05 (for 12 tests performed)

*Abbreviations*: PD: Parkinson’s disease; PD-F: PD fallers; PD-NF: PD non-fallers; SD: standard deviation; CI: confidence interval; OFF: “off” medication state; ON: “on” medication state; UPDRS-III: Unified Parkinson Disease Rating Scale, Motor score; Presence of dyskinesia: number of patients with UPDRS IV A ≠ 0; Dyskinesia subscore: sum of UPDRS items 32 and 33; GBS: Gait and balance score; NMS-30: Non-motor symptom scale; MoCA: Montreal Cognitive Assessment; FAB: Frontal Assessment Battery; BDI-II: Beck Depression Inventory, Second Edition; STAI: State-Trait Anxiety Inventory (X1 = State anxiety, X2 = Trait anxiety); FES-I: Short Falls Efficacy Scale-International; FOG: Freezing of gait, UPDRS-II, item 14; ESSOT: Composite equilibrium score in Sensory organization test; CV: coefficient of variation

Considering medication-induced changes, UPDRS-III and GBS scores significantly decreased in the ON compared to OFF state in both PD-F and PD-NF groups. In addition, in PD-F, walking speed and stride time variability improved in the ON state. In PD-NF, only walking speed improvement reached statistical significance. The other parameters measured remained unchanged in both groups between ON and OFF states (see **Table 2**).

**Fig 2:**
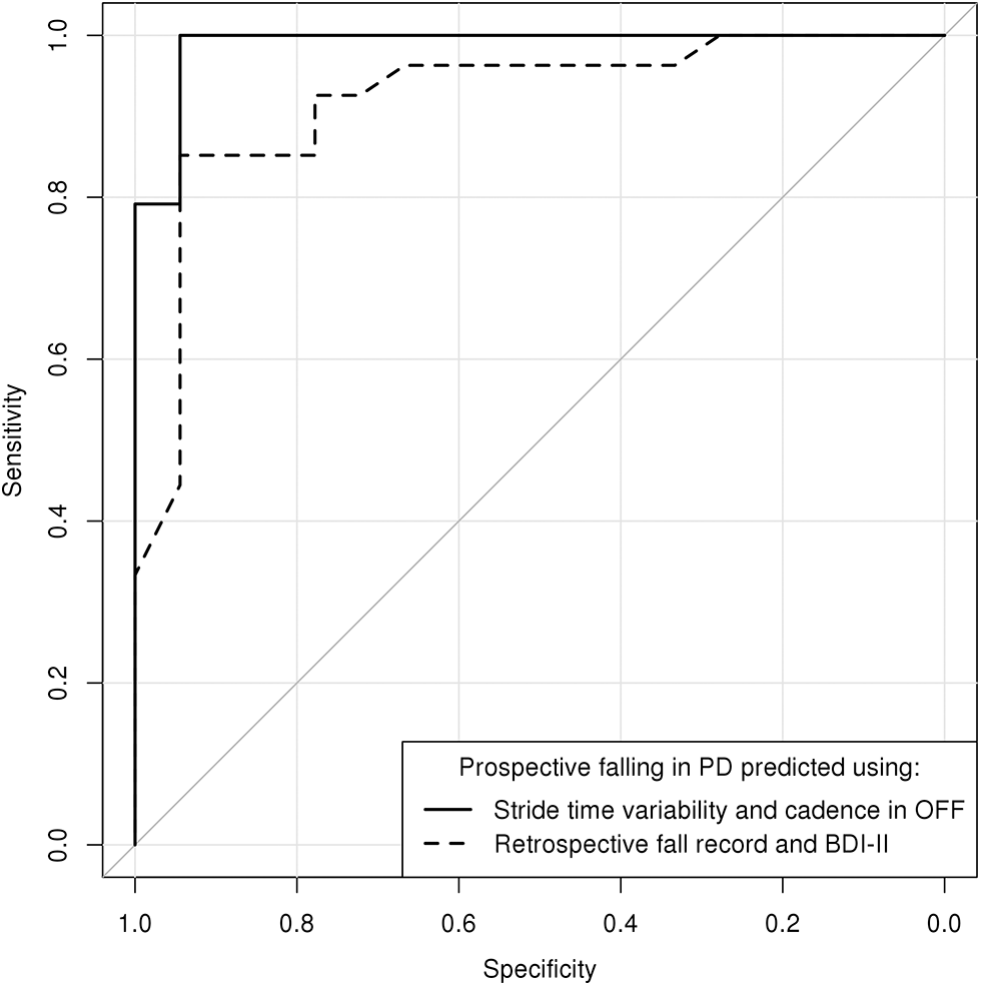
Receiver operating characteristic (ROC) of two predictive models of prospective falls in PD patients. Solid curve: The instrumental model incorporating stride time variability and cadence in the OFF state. Dashed curve: The clinical model incorporating retrospective fall record and BDI-II

The set of candidate clinical predictors for the multiple logistic regression model of prospective falling included the predictors identified as highly relevant by univariate models (Table 2, p<0.001 uncorrected): retrospective fall record, HY stage, expressed fear of falling (FES-I), depressive symptoms (BDI-II) and GBS in the OFF state. The most relevant parameters were revealed to be the retrospective fall record and BDI-II, jointly reaching an AIC of 34.1, BIC of 39.3, an area under the ROC curve (AUC) of 0.921, sensitivity of 85% and specificity of 94% (**Table 3**, **Fig 2**). Candidate instrumented predictors included all measured instrumental parameters: stride time variability, cadence, gait velocity and ESSOT (all in the OFF state, as we could not use parameters in both the OFF and ON states due to colinearity, and OFF parameters seemed to be more informative compared to ON parameters). Stride time variability and cadence, combined in a single model, were found to be the most relevant, reaching an AIC of 14.9, BIC of 20.1, an AUC of 0.988, sensitivity of 100% and specificity of 94% (**Table 3, Fig 2**). The model predicted a patient to be faller if 7.2 × stride time variability in the OFF state + 0.5 × cadence in the OFF state was greater than 80 (**Fig 1B**). Predictive performance of the instrumented model was not improved by incorporating parameters from the clinical model (likelihood ratio test P>0.1 for each of the retrospective fall record and BDI-II covariates), however the performance of the clinical model was significantly increased by incorporating stride time variability in the OFF state as an additional covariate (χ^2^ = 7.77, df = 1, P<0.001), leading to a combined model that could neither be improved nor simplified, reaching an AIC of 15.8, BIC of 22.8, an AUC of 0.995, sensitivity of 96% and specificity of 100%. As both the AIC and BIC were in favor of the instrumented model against the combined model, and given the limited number of patients in the present study, we preferred the instrumented model (based on only two covariates) over the combined model (based on three covariates) to overcome overfitting, and selected the instrumented model to be the final multiple logistic regression model of prospective falling.

**Table 3:** Multiple logistic regression models of (prospective) falling in PD patients based on clinical and instrumental parameters.

Covariate	Covariate Change Associated with the Odds Ratio	Odds Ratio	95% CI	p-value
**Clinical model**				
Retrospective fall status	1	42	(7–508)	<0.001
BDI-II	1	1.3	(1.1–1.7)	<0.01
**Instrumental model**				
Stride time variability OFF (CV%)	0.1	2.1	(1.3–5.9)	<0.001
Cadence OFF (steps/min)	1	1.7	(1.2–4.3)	<0.001

Odds ratios are associated with respective change in each covariate, conditionally on the other covariate kept fixed.

P-values are based on likelihood ratio test comparing the full model with a submodel with the respective covariate removed.

*Abbreviations*: CI: confidence interval; OFF: “off” medication state; BDI-II: Beck Depression Inventory, Second Edition; CV: coefficient of variation

In addition, post hoc analyses revealed significant association between stride time variability and total BDI-II score (r = 0.45, P<0.05 corr.) and with the sum of UPDRS gait items 15 and 29 (r = 0.46, P<0.05 corr.) in the OFF state. Stride time variability was not related to any other parameter (**S2 Table**).

## Discussion

In the present study, we primarily aimed to verify the utility of demographic, disease specific clinical, gait and balance related parameters recorded in both OFF and ON medication states in the prediction of future falls in patients with moderate stage PD.

Several notable differences were found between prospectively identified PD fallers and non-fallers. Similarly to previous studies [5, 6, 8, 9, 13], PD fallers had a significantly greater number of retrospective falls, greater disease severity, higher daily dose of dopaminergic medication, higher scores in self-rating questionnaires of depression and fear of falling and more severe motor impairment, in particular a higher GBS score in the OFF state compared to PD non-fallers. In addition, from the instrumented gait and balance examination, stride time variability in the OFF medication state differed between both PD subgroups while other gait and balance measures failed to show any significant differences. In a multiple logistic regression model of prospective falling based on clinical parameters, a history of falls and depressive symptoms (BDI-II) had the highest discriminative value in identifying future fallers, while the combination of stride time variability and cadence in the OFF medication state was the best instrumented predictor of future falls. Note that even though cadence alone could not predict falling, it contributed significant additional information on top of stride time variability to the instrumental model, attributing high risk of falls to patients experiencing high stride time variability or high cadence, or a high sum of these (**Fig 1B**).

Previous studies have reported that gait variability is increased among PD patients in general [33–35] and in retrospective PD fallers [21]. Here, we extend these results and demonstrate that stride time variability may predict falls in prospectively identified PD fallers. In addition, we found that stride time variability correlated with the total BDI-II score, which was clearly increased in our PD fallers. This is consistent with studies showing that depressive symptoms are associated with gait dysfunction and falls in PD patients [36] as well as in cognitively intact older adults [37]. Notably, depression is a potentially remediable risk factor for falling, although currently there is no evidence that the improvement of depression is associated with a reduction of falls in PD patients [38].

Conversely, we could not confirm the predictive value of several factors identified previously, namely FOG, dyskinesia and global cognitive and executive function. The ability to regulate stride-to-stride variability and maintain a stable walking rhythm is markedly impaired in subjects with FOG [39], which may contribute to falls. Although the incidence of FOG was low in our cohort, gait variability was markedly increased in prospective PD fallers. Furthermore, several studies reported that the presence of dyskinesia is related to likelihood of falls [40–42]. We did not find association between dyskinesia and future falls, even if incidence of dyskinesia was insignificantly higher in our PD fallers group compared to PD non-fallers. It is consistent with other recent prospective studies [7, 9], which did not demonstrate association between dyskinesia and faller status. Finally, a link between cognitive performance, gait disorders and falls has been suggested [43] but several prospective studies did not identify global cognitive impairment or executive function as risk factors for falling [7, 9, 19, 44]. Indeed, neither general cognitive (MoCA) nor frontal (FAB) dysfunction was related to stride time variability and risk of falls in our patients.

Our results favor testing PD patients in the OFF state, in which future fallers showed significantly higher GBS scores and stride time variability compared to non-fallers. Also, according to the mathematical model, OFF state stride time variability and cadence appear to be suitable markers for detecting future fallers. These results are in agreement with Foreman et al [17] and are strengthened by the fact that our study had a prospective design.

Finally, by comparing initial patient performance in the ON and OFF medication states, we can estimate the respective contribution of dopaminergic and nondopaminergic factors to gait and balance involvement and to falls in PD. Beside its general effect on motor performance, levodopa produced improvements in walking speed and stride time variability in our cohort. This is consistent with previous studies that demonstrated improvements both in walking speed and stride time variability with dopaminergic medication in PD [21, 36, 45, 46], suggesting possible therapeutic implications in PD fallers.

There are some limitations to the present study. Due to restricted time for testing in the ON state, only a limited number of clinical and instrumented tests could be performed to ensure that patients remained in a stable motor state. Fixed order of testing in the OFF followed by the ON state was the only way to perform testing in one day, to provide for otherwise identical conditions. Thus, a practice effect could have influenced ON state performance, however this would not invalidate the results obtained in the OFF state. Furthermore, due to the relatively small number of participants, we could not include more candidate predictors of future falls in the logistic regression model. It was also not possible to reliably distinguish whether falls occurred in the OFF or ON states in the prospective follow-up. Although information on falls was gathered using fall diaries and telephone interviews as commonly done in previous studies [5, 6], the documentation of falls may suffer from recall bias.

In summary, the present study shows that a history of previous falls, fear of falling, higher disease stage, levodopa dose, and more severe motor impairment in the OFF state represent important risk factors for future falls in PD patients. In addition, instrumented testing of gait in the OFF state demonstrated increased stride time variability in combination with faster walking cadence as the most significant predictors of future falls in PD patients. While instrumented testing in the OFF state is not easily implemented in everyday clinical practice, advances in technology may help to more effectively address the risk of falling in PD patients and to translate results into preventive measures [47]. Namely, body worn sensors allowing daylong monitoring of gait, balance and falls provide the opportunity for immediate biofeedback that can focus patient attention and enhance performance [48].

## Supporting Information

S1 File Detailed instrumented evaluation of balance and gait. (DOCX)

S1 Table Detailed classification of prospective falls. (DOCX)

S2 Table Relationship between stride time variability and baseline parameters. (DOCX)
