# Correction: Predicting Falls in Parkinson Disease: What Is the Value of Instrumented Testing in OFF Medication State?

**DOI:** 10.1371/journal.pone.0142765

**Published:** 2015-11-06

**Authors:** The PLOS ONE Staff

There are errors in the Funding section. The complete, correct Funding Statement is as follows: This study was supported by the Czech Ministry of Health, NS10336-3/2009, http://iga.mzcr.cz/publicWeb/, MH; Czech Ministry of Health, NT11190-6/2010, http://iga.mzcr.cz/publicWeb/, HB; Czech Ministry of Health, NT12282-5/2011,http://iga.mzcr.cz/publicWeb/, RJ; Czech Ministry of Health, NT14181-3/2013, http://iga.mzcr.cz/publicWeb/, ER; Charles University in Prague, PRVOUK P26/LF1/4,. http://is.cuni.cz/webapps/whois2/org/1365292082243020/, ER; European social fund realized at the Czech Technical University in Prague, CZ.1.07/2.3.00/30.0034, TS.

The publisher apologizes for the errors.
