# Supplementary material for: Predicting Falls in Parkinson Disease: What Is the Value of Instrumented Testing in OFF Medication State?
Source: PLoS One. 2015 Oct 7;10(10):e0139849. doi: 10.1371/journal.pone.0139849 (PMC4596567; doi:10.1371/journal.pone.0139849)
Supplement: S1 File — (DOCX) [file pone.0139849.s001.docx]

**Predicting falls in Parkinson disease: What is the value of instrumented testing in OFF medication state?**

**Supporting information**

**Patients and methods**

*Instrumented gait and balance assessment*

Gait analysis was performed using portable inertial sensors (Xsens MTx; Enschede, the Netherlands) in the instrumented Timed Up and Go (iTUG) test extended from traditional 3 m (traditional TUG) to 7 m to provide enough steps for gait analysis according to Zampieri et al. (1). Three walking trials were performed. Five sensors were fixed by elastic bands on the lateral sides of the legs just above the ankles, on dorsa of the wrists and on the sternum. For the analysis presented here, only data from the legs were used. All subjects were instructed to stand up from a chair, walk forward at their self-paced velocity 7 m to a horizontal yellow line on the floor, turn around, walk back and sit down. Individual strides were detected only during straight walk and the average values from two straight distances (both distances was 7m long) within second trial were used to data analysis. The following gait outcomes representing general mobility and gait variability were collected: (1) Gait speed (m/s); (2) Cadence (steps/min) and (3) Stride time variability which was chosen as a potential predictor of falls in PD patients (2). Stride time variability was calculated as the coefficient of variation (CV) of stride durations, i.e. as the ratio of the standard devoation to the mean stride duration. All the outcomes were calculated from gait events (initial and terminal contacts) detected using angular speeds of the lower legs and algorithm as described by Salarian et al. (3). For calculation of the gait speed, also stride lengths calculated using an estimation algorithm from the angular speeds published by Moore et al. were used (4). Balance was evaluated by means of computerized dynamic posturography (The Smart Balance Master®; Neurocom, Clackamas, Oregon, USA) using a composite equilibrium score in sensory organization test (ESSOT). The sensory organization test (SOT) measures a subject’s postural sway in six conditions of stationary or moving support surfaces with appropriate, inappropriate, or no visual feedback. Subjects were secured in a safety harness. For each SOT condition, the subject’s sway is represented by the equilibrium score (ES), which compares the subject sway with the theoretical limit of stability. The ES for each condition reflects the average score for three 20-second trials (5).

References:

1. Zampieri C, Salarian A, Carlson-Kuhta P, Aminian K, Nutt JG, Horak FB. The instrumented timed up and go test: potential outcome measure for disease modifying therapies in Parkinson's disease. Journal of neurology, neurosurgery, and psychiatry. 2010;81(2):171-6.

2. Schaafsma JD, Giladi N, Balash Y, Bartels AL, Gurevich T, Hausdorff JM. Gait dynamics in Parkinson's disease: relationship to Parkinsonian features, falls and response to levodopa. Journal of the neurological sciences. 2003;212(1-2):47-53.

3. Salarian A, Russmann H, Vingerhoets FJ, Dehollain C, Blanc Y, Burkhard PR, et al. Gait assessment in Parkinson's disease: toward an ambulatory system for long-term monitoring. IEEE transactions on bio-medical engineering. 2004;51(8):1434-43.

4. Moore ST, MacDougall HG, Gracies JM, Cohen HS, Ondo WG. Long-term monitoring of gait in Parkinson's disease. Gait & posture. 2007;26(2):200-7.

5. Bronte-Stewart HM, Minn AY, Rodrigues K, Buckley EL, Nashner LM. Postural instability in idiopathic Parkinson's disease: the role of medication and unilateral pallidotomy. Brain : a journal of neurology. 2002;125(Pt 9):2100-14.
