# Supplementary material for: Predicting Falls in Parkinson Disease: What Is the Value of Instrumented Testing in OFF Medication State?
Source: PLoS One. 2015 Oct 7;10(10):e0139849. doi: 10.1371/journal.pone.0139849 (PMC4596567; doi:10.1371/journal.pone.0139849)
Supplement: S1 Table — (DOCX) [file pone.0139849.s002.docx]

| **Prospective fall rates** | **PD** (N=45) | **NC** (N=22) |
| --- | --- | --- |
| Fall frequency | 185 | 5 |
| Time to first fall (months) | 2.2±1.6 | 3.8±1.3 |
| Fallers | 27 (60.0%) | 4 (18.2%) |
| Falls indoors | 133 (71.9%) | 1 (20%) |
| Falls outdoors | 52 (28.1%) | 4 (80%) |
| Falls in forward direction | 93 (50.3%) | 4 (80%) |
| Falls in backward direction | 40 (21.6%) | 0 (0%) |
| Falls in lateral direction | 52 (28.1%) | 1 (20%) |
| Falls with injuries (without fractures) | 3 (1.6%) | 0 (0%) |
| Falls with fractures | 2 (1.1%) | 0 (0%) |
| Lach classification (Lach 1991) |  |  |
| Intrinsic factors | 125 (67.6%) | 1 (20%) |
| Extrinsic factors | 31 (16.8%) | 3 (60%) |
| Non-bipedal stance | 3 (1.6%) | 0 (0%) |
| Unclassified falls | 26 (14.1%) | 1 (20%) |
| Maki classification (Maki 1994) |  |  |
| Base of support falls | 30 (16.2%) | 3 (60%) |
| Centre of mass falls: | 126 (68.1%) | 1 (20%) |
| FOG and festination | 19 (10.3%) | NA |
| Bending forward | 34 (18.4%) | 1 (20%) |
| Bending laterally | 4 (2.2%) | 0 (0%) |
| Standing up | 21(11.4%) | 0 (0%) |
| Turning around | 48 (26.0%) | 0 (0%) |
| No obvious perturbation falls | 10 (5.4%) | 0 (0%) |
| Unclassified falls | 19 (10.3%) | 1 (20%) |

**S1 Table. Detailed classification of prospective falls**

*Abbreviations*: PD: Parkinson’s disease patients; NC: normal controls; FOG: freezing of gait; NA: not available

Data are displayed as mean±standard deviation or as individual counts and percentage in parentheses.
