# Supplementary material for: Predicting Falls in Parkinson Disease: What Is the Value of Instrumented Testing in OFF Medication State?
Source: PLoS One. 2015 Oct 7;10(10):e0139849. doi: 10.1371/journal.pone.0139849 (PMC4596567; doi:10.1371/journal.pone.0139849)
Supplement: S2 Table — (DOCX) [file pone.0139849.s003.docx]

| **Domains and parameters** | **Stride time variability** | |
| --- | --- | --- |
|  | OFF state | ON state |
| **Demographic and disease specific variables** | | |
| Age (yrs) | -0.06 (0.728) | 0.26 (0.085) |
| PD duration (yrs) | 0.09 (0.579) | -0.10 (0.510) |
| Hoehn and Yahr stage | 0.31 (0.046) | 0.16 (0.304) |
| UPDRS- II+III | 0.28 (0.070) | 0.10 (0.512) |
| Tremor (items 20 + 21) | 0.05 (0.759) | -0.18 (0.228) |
| Rigidity (item 22) | 0.11 (0.483) | -0.01 (0.959) |
| Body bradykinesia (item 31) | 0.32 (0.038) | 0.18 (0.231) |
| Falls (item 13) | 0.37 (0.015) | 0.30 (0.044) |
| Freezing (item 14) | 0.05 (0.761) | 0.07 (0.664) |
| Gait (items 15 + 29) | 0.46 (0.002)* | 0.37 (0.012) |
| **Cognition, anxiety** | | |
| MoCA | -0.18 (0.261) | -0.20 (0.183) |
| FAB | -0.34 (0.027) | -0.35 (0.019) |
| BDI-II | 0.45 (0.003)* | 0.18 (0.246) |
| STAI X1 | 0.20 (0.213) | 0.15 (0.331) |
| STAI X2 | 0.21 (0.172) | -0.04 (0.799) |

**S2 Table. Relationship between stride time variability and baseline parameters**

* differences significant at the Holm-Bonferroni-corrected level of p < 0.05 (for 16 tests performed in each of the OFF and ON states).

Table entries are Pearson correlation coefficients (P-values).

*Abbreviations*: UPDRS-II+III: Unified Parkinson’s Disease Rating Scale, Activities of daily life and Motor score; MoCA: Montreal Cognitive Assessment; FAB: Frontal Assessment Battery; BDI-II: Beck Depression Inventory, Second Edition; STAI: State-Trait Anxiety Inventory (X1 = State anxiety, X2 = Trait anxiety); FES-I: Short Falls Efficacy Scale-International;
